# Functional Genomic mRNA Profiling of a large cancer data base demonstrates mesothelin overexpression in a broad range of tumor types

**DOI:** 10.18632/oncotarget.4461

**Published:** 2015-07-01

**Authors:** Laetitia E. Lamberts, Derk Jan A. de Groot, Rico D. Bense, Elisabeth G.E. de Vries, Rudolf S.N. Fehrmann

**Affiliations:** ^1^ University of Groningen, University Medical Center Groningen, Department of Medical Oncology, Groningen, The Netherlands

**Keywords:** mesothelin, drug target, functional genomic mRNA profiling, overexpression, antibody-drug conjugate

## Abstract

The membrane bound glycoprotein mesothelin (MSLN) is a highly specific tumor marker, which is currently exploited as target for drugs. There are only limited data available on MSLN expression by human tumors. Therefore we determined overexpression of *MSLN* across different tumor types with Functional Genomic mRNA (FGM) profiling of a large cancer database. Results were compared with data in articles reporting immunohistochemical (IHC) MSLN tumor expression. FGM profiling is a technique that allows prediction of biologically relevant overexpression of proteins from a robust data set of mRNA microarrays. This technique was used in a database comprising 19,746 tumors to identify for 41 tumor types the percentage of samples with an overexpression of *MSLN* compared to a normal background. A literature search was performed to compare the FGM profiling data with studies reporting IHC MSLN tumor expression. FGM profiling showed *MSLN* overexpression in gastrointestinal (12–36%) and gynecological tumors (20–66%), non-small cell lung cancer (21%) and synovial sarcomas (30%). The overexpression found in thyroid cancers (5%) and renal cell cancers (10%) was not yet reported with IHC analyses. We observed that *MSLN* amplification rate within esophageal cancer depends on the histotype (31% for adenocarcinomas versus 3% for squamous-cell carcinomas). Subset analysis in breast cancer showed *MSLN* amplification rates of 28% in triple-negative breast cancer (TNBC) and 33% in basal-like breast cancer. Further subtype analysis of TNBCs showed the highest amplification rate (42%) in the basal-like 1 subtype and the lowest amplification rate (9%) in the luminal androgen receptor subtype.

## INTRODUCTION

Mesothelin (MSLN) is a membrane bound glycoprotein with only limited expression in normal tissues such as mesothelial cells lining pleural, pericardial and peritoneal surfaces [1]. This makes it an interesting target for anticancer drugs. Its function is largely unknown. In mice inactivation of the MSLN gene produced physiologically normal, fertile offspring without any anatomical or histological abnormalities. This demonstrated no essential role for MSLN for growth in mice [2]. Studies with immunohistochemical (IHC) analyses showed high MSLN expression in 100% of the epithelial mesotheliomas, 90–100% of pancreatic and 66–100% of ovarian cancers. This is of interest as these tumors largely lack targets for targeted agents. MSLN is also known to be overexpressed to a lesser extent in multiple other human cancers such as endometrial, lung, stomach, triple negative breast, cervical, non-small cell lung cancer (NSCLC) and head and neck cancers (HNSCC) [3–8].

Increasing insight in tumor biology has accelerated the development of molecularly targeted drugs. Many of these drugs target molecular drivers of tumor growth with the goal to inhibit their downstream effects in a tumor cell. In contrast, novel drugs are becoming available that target over-expressed tumor specific antigens such as MSLN that have no clear role in tumor genesis. Among these novel drugs are the antibody-drug conjugates (ADCs), which combine the specific targeting of an antibody with the potency of cytotoxins that would alone cause severe dose-limiting toxicities [9–11]. Critical for ADC efficacy is overexpression of a target antigen at the cell membrane of tumor cells. After internalization the toxic load is activated.

The same mechanism of action is exploited by immunotoxins, which consist of a targeting antibody (fragment) fused with a toxin. An interesting example targeting MSLN is the immunotoxin SS1P, comprising a portion of a *Pseudomonas* exotoxin [12].

Another strategy targeting tumor cells overexpressing a certain antigen is immunotherapy. An example are the cancer vaccines such as GVAX, a combination of two irradiated, granulocyte-macrophage colony stimulating factor secreting allogeneic pancreatic cancer cell lines which were administered to patients with irresectable or metastasized pancreatic cancer. The cancer cell lines were combined with recombinant live-attenuated, double-deleted *Listeria* monocytogenes, engineered to secrete MSLN into the cytosol of infected antigen presentation cells. The combination of these two agents induces an *in vivo* immune response to mesothelin expressing pancreatic cancer cells [13].

Additionally, chimeric antigen receptor (CAR)-engineered T cells using MSLN as a target are developed as adoptive T cell immunotherapy in patients [14]. Three clinical trials are ongoing (NCT01355965, NCT01583686, NCT02159716) and two partial responses (PR) were already reported; one in a patient with pancreatic cancer and one patient with malignant pleural mesothelioma [15].

Although several IHC studies are performed evaluating percentages of MSLN overexpression, these numbers may not reflect the actual percentages of tumors with useful MSLN expression as they are based on small numbers of tumors per type, different assays and different definitions of positivity.

We have recently developed a method called functional genomic mRNA (FGM) profiling that corrects gene expression data (*i.e.* mRNA expression data) for major, non-genetic, factors (*e.g.* physiological, metabolic, cell-type-specific and experimental factors) [16]. We observed that the residual gene expression signal (*i.e.* FGM profile) correlated strongly with somatic copy number alterations (SCNAs) in cancer samples. In other words, with FGM profiling we are capturing the downstream effect of SCNAs at gene expression levels. FGM profiling is particularly useful because of the public availability of microarray expression profiles for thousands of cancer samples. We applied this method to publicly available expression data of 19,746 unrelated, patient-derived tumor samples to gain more detailed information about the position of MSLN as a generalizable drug target in 41 tumor types and compared this data to currently existing IHC data from literature.

## RESULTS

### Mesothelin expression analyzed by FGM profiling

**Figure 1:**
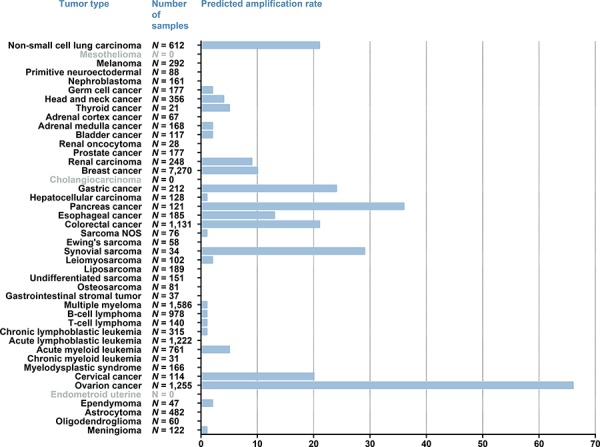
MSLN overexpression calculated with FGM profiling Values adjacent to the bars represent the absolute number of tumors analyzed per tumor type. The x-axis represents the predicted percentage of samples per tumor type that show an overexpression of MSLN. For the tumor type indicated in grey no FGM profiles were available.

The median number of samples per tumor type was 161, ranging from 21 for thyroid cancer to 7,270 for breast cancer. The number of samples per tumor type in combination with the predicted percentage of samples with a *MSLN* amplification is shown in Fig. 1.

Predicted amplification of *MSLN* was most frequently found in gynecological tumors, gastrointestinal tumors, NSCLC (21% of *N* = 612) and in synovial sarcoma (30% of *N* = 34). In ovarian cancer 66% of 1,255 tumors had a predicted *MSLN* amplification and in cervical cancers (*N* = 114) this was 20%. Highest predicted *MSLN* amplification rate for gastrointestinal cancer was seen in pancreatic adenocarcinomas (36% of *N* = 121), followed by gastric cancers (24% of *N* = 212) and colorectal cancers (21% of *N* = 1,131). A predicted *MSLN* amplification rate of 13% was seen for esophageal cancer (*N* = 185), which was mainly driven by the subset of esophageal adenocarcinomas with an *MSLN* amplification rate of 31% (*N* = 64). In contrast, for esophageal squamous-cell carcinomas only a *MSLN* amplification rate of 3% (*N* = 109) was observed.

Additionally, predicted *MSLN* amplifications were found in 9% of the renal cell carcinomas (*N* = 428 tumors), 5% of the thyroid cancers (*N* = 21 tumors), 5% of acute myeloid leukemias (*N* = 761 tumors) and 4% of the HNSCC (*N* = 356 tumors).

We observed a predicted *MSLN* amplification rate of 10% in the total set of breast cancer samples (*N* = 7,270). Within the subset of estrogen receptor (ER) positive (*N* = 4,906) and within the subset of human epidermal growth factor 2 (HER2) positive (*N* = 1,580) breast cancer samples the *MSLN* amplification rate was 3% and 7%, respectively (Table 1). The observed MSLN amplification rate within the subset of TNBC samples (*N* = 1,555) was 28%. Within the subset of breast cancer samples for which we were informed on the molecular subtype classification, we observed a high *MSLN* amplification rate within the basal-like subtype (33% of *N* = 378). After applying the TNBC sub-classification according to Lehman et al. on the subset of TNBC samples we observed the highest *MSLN* amplification rate (42%) within the basal-like 1 class (*N* = 282). The lowest amplification rate (9%) was observed for the luminal androgen receptor class (*N* = 164).

**Table 1:** Predicted MSLN amplification rate in breast cancer subtypes

**Subset**	**MSLN negative (*n*)**	**MSLN negative (%)**	**MSLN positive (*N*)**	**MSLN positive (%)**	**Total (*N*)**
TNBC	1114	71.64	441	28.36	1555
non-TNBC	5459	95.52	256	4.48	5715
ER-positive	4741	96.64	165	3.36	4906
HER2-positive	1472	93.16	108	6.84	1580
Subset^*1^	**MSLN negative (*N*)**	**MSLN negative (%)**	**MSLN positive (*N*)**	**MSLN positive (%)**	**Total (*N*)**
Normal-like	118	98.33	2	1.67	120
Luminal A	326	99.69	1	0.31	327
Luminal B	154	94.48	9	5.52	163
Her2	118	93.65	8	6.35	126
Basal	254	67.20	124	32.80	378
Subset^*2^	**MSLN negative (*n*)**	**MSLN negative (%)**	**MSLN positive (*N*)**	**MSLN positive (%)**	**Total (*N*)**
Basal-like 1	164	58.16	118	41.84	282
Basal-like 2	98	76.56	30	23.44	128
Mesenchymal	156	63.41	90	36.59	246
Mesenchymal stem–like	130	87.84	18	12.16	148
Immunomodulatory	226	71.97	88	28.03	314
Luminal androgen receptor	150	91.46	14	8.54	164

*1 Molecular sub-classification according to methods: Hu *et al*. - Parker *et al*. - Sorlie *et al*. [30–32]

*2 TNBC subclassification according to Lehmann *et al*. [33]

### Mesothelin expression measured immunohistochemically in published papers

We identified 14 published papers in which IHC staining for MSLN was described for a total of 2,846 tumor samples [1, 5–8, 17–26]. The number of samples analyzed per tumor type ranged from 3 (gastrointestinal stromal tumors) to 1,209 samples (NSCLC) with a median of 20 per tumor type. IHC was performed with a total of 5 different anti-MSLN staining antibodies and 13 different scoring systems. The most frequently used antibody (9 out of the 15 studies) is the 5B2 monoclonal antibody from Novocastra [5, 18–21, 24, 25, 27]. Other staining antibodies were K1,5B2 from Thermo-scientific, 5B2 from Vector Laboratories, 22A31 and K1 [1, 7, 8, 22, 23]. For all 15 studies, 13 different scoring strategies were applied. Only the Ordonez papers [18, 19] used the same scoring system, and also Kachala [6] and Tozbikian [8] used the same method.

The most MSLN over-expressing tumor types were synovial sarcoma (100%) ovarian (50–88%), NSCLC, endometrioid uterine adenocarcinoma (59–64%), cervical (25%), pancreatic (86–100%), colorectal (28–50%), esophageal (25–46%) and gastric carcinoma (27–58%) [5, 15, 17, 18, 20–24]. Percentages of breast cancer with MSLN overexpression varied between studies, probably due to different subtypes being analyzed. In *N* = 43 triple negative breast cancer (TNBC) samples, 67% was MSLN positive, while in other sub types comprising 29 ER positive and 27 HER2 positive, this was below 5% [7]. Others reported a high MSLN expression in 36% of TBNC samples, compared to 16% in non-TNBC samples [8]. In Table 2 IHC MSLN expression data are shown per tumor type.

**Table 2:** Individual MSLN immunohistochemistry studies published

Tumor type	% positive	N*	Reference^£^
Non-small cell lung carcinoma Non-small cell lung carcinoma Non-small cell lung carcinoma Non-small cell lung carcinoma	43% 41% 0% 69%	47 34 23 1209	5 18 1 6
Mesothelioma Mesothelioma	100% 100%	15 40	1 19
Melanoma	0%	6	18
Primitive neuroectodermal tumor	0%	6	18
Germ cell cancer	0%	23	18
Thyroid cancer Thyroid cancer	3% 0%	29 14	5 18
Adrenal cortex cancer	0%	5	18
Bladder cancer Bladder cancer	0% 8%	8 13	5 18
Prostate cancer Prostate cancer	1% 0%	85 12	5 18
Renal carcinoma Renal carcinoma	3% 0%	33 17	5 18
Breast cancer Breast cancer Breast cancer Breast cancer Breast cancer	14% 67% 28% 36% 3%	71 43 80 226 35	5 7* 19 8* 18
Cholangiocarcinoma Cholangiocarcinoma Cholangiocarcinoma Cholangiocarcinoma	100% 37% 50% 95%	61 19 10 21	20 18 23 3
Gastric cancer Gastric cancer Gastric cancer Gastric cancer	29% 45% 27% 58%	7 110 156 50	18 24 5 23
Hepatocellular carcinoma	0%	15	5
Pancreas cancer Pancreas cancer Pancreas cancer Pancreas cancer	86% 100% 83% 100%	14 14 60 16	18 5 17 3
Esophageal cancer Esophageal cancer Esophageal cancer	27% 25% 46%	156 4 125	5 18 22
Colorectal cancer Colorectal cancer Colorectal cáncer	50% 30% 28%	91 56 18	21 5 18
Ewing's sarcoma	0%	6	18
Synovial sarcoma	100%	9	18
Leiomyosarcoma	0%	5	18
Gastrointestinal stromal tumor	0%	3	18
B-cell lymphoma	0%	8	18
T-cell lymphoma	0%	8	18
Cervical cancer	25%	4	18
Ovarian cancer Ovarian cancer Ovarian cancer	70% 55% 88%	67 198 40	5 26 18
Endometroid uterine adenocarcinoma Endometroid uterine adenocarcinoma	59% 64%	22 11	5 18
Head and Neck cancer	67%	6	1

* N is the number of tumor samples analyzed in the study.

£ Percentage positive is the percentage of all tumors analyzed (in column *N*) that were mesothelin positive, according to the original article.

### MSLN overexpression by functional genomic mRNA profiling versus IHC

The patterns of MSLN overexpression are largely comparable between the historical IHC data and our data gathered with FGM profiling. The percentages of tumor samples that showed MSLN over-expression or predicted *MSLN* amplification differ between the two techniques, with on average higher percentages for the IHC data. For example, NSCLC shows in 69% of tumors an over-expression based on IHC, while we find a predicted *MSLN* amplification rate for NSCLC of 21%. The same is true for the synovial sarcomas, colorectal, pancreatic and gastric cancers. This does not account for all tumors, as 50–88% of ovarian cancers are considered MSLN positive based on IHC, while with our technique we find 66% of ovarian cancer samples having a predicted *MSLN* amplification.

For renal cell and thyroid cancer, IHC studies were performed, although in small numbers of tumors (*N* = 33 for renal cell, and *N* = 14 and *N* = 29 for thyroid cancer).

## DISCUSSION

This is the first paper studying MSLN expression in a large database of human tumors with a novel method called FGM profiling. We showed high percentages of predicted *MSLN* amplification in gynecological tumors, gastrointestinal tumors, NSCLC and synovial sarcomas. In addition, our technique revealed not yet reported predicted *MSLN* amplifications in 10% of renal cell cancers and 5% of thyroid cancers. In addition, we observed that *MSLN* amplification rate within esophageal cancer depends on the histotype (31% for adenocarcinomas versus 3% for squamous-cell carcinomas). Subtype analysis in breast cancer showed *MSLN* amplification rates of 28% in triple-negative breast cancer (TNBC) and 33% in basal-like breast cancer. Within the TNBCs the basal-like 1 subtype showed the highest amplification rate (42%) and the luminal androgen receptor subtype the lowest amplification rate (9%).

This data suggests which percentages of tumor types potentially might benefit from treatment with MSLN targeting immunotoxins or ADCs. For mesothelioma, pancreatic and ovarian cancer, drugs are currently in development that target MSLN [3, 12, 15, 28, 29]. However, also for the lower percentages of MSLN over-expressing tumors within other tumor types over-expression of MSLN could be an interesting target. In this era of individualized treatment, tumor histology types alone do not have to determine which therapy will be most effective. Increasingly, drug development focuses on tumor characteristics and targeting these, than on tumor type alone.

IHC is most often applied for a semi-quantitative analysis of protein expression in tumor samples. However, IHC studies have well-known disadvantages, the first being highly heterogeneous scoring methods between different studies. For example, Frierson et *al*. classified MSLN protein expression as 1+ when only 1–10% of tumor cells showed positive staining, while others use a combination of staining intensity and percentage of positive stained tumor cells [5–8, 18, 20–26]. Moreover, different staining antibodies have been used in the different studies. This makes it currently difficult to compare IHC patterns in different studies of different tumor types. Also it precludes a general cut off for IHC indicating over-expression of MSLN. If a relevant target, standardization of IHC for MSLN would be clearly required. FGM profiling provides a rapid screening tool for potentially drugable targets in a large set of tumors, but also has some drawbacks. No quantitative analysis is possible and there is no direct correlation between the FGM profile and protein levels of the genes investigated. Moreover, it is not possible determine heterogeneity in expression between tumor cells or to determine which cell type in the tumor tissue expresses MSLN.

The advantages of FGM profiling however prevail and include that predicted *MSLN* amplification rates between tumor types are directly comparable as the same threshold is used. In addition, the large number of samples included in this analysis and the broad spectrum of tumor types allow for robust estimations of predicted *MSLN* amplification rates. FGM profiling may also be useful in determining over-expression of other potentially drugable targets in different tumor types. This highly facilitates prioritization of tumor types for future research in which the clinical benefit of targeting MSLN with immunotoxins or ADCs.

## MATERIALS AND METHODS

### Functional genomic mRNA profiling

For a detailed description of FGM profiling we refer to Fehrmann *et al*. [16]. In short, we analyzed 77,840 expression profiles of publicly available samples with principal component analysis (PCA) and found that a limited number of ‘Transcriptional Components’ (TCs) capture the major regulators of the mRNA transcriptome. Subsequently, we identified a subset of TCs that described non-genetic regulatory factors. We used these non-genetic TCs as covariates to correct microarray expression data and observed that the residual expression signal (*i.e.* FGM profile) captures the downstream consequences of genomic alterations on gene expression levels.

### Identification of 19,746 unrelated, patient-derived tumor samples

As described in more detail in Fehrmann *et al*, we were able to construct a set of 15,878 unrelated tumor samples of patients. In short, each of the 77,840 samples was annotated with MeSH terms based on an automatic text-mining algorithm. Next, we developed a method to exclude cell line samples, as these samples might not reflect the *in vivo* situation of cancer cells. Subsequently, we developed a method that can accurately detect and exclude genetically identical samples in expression data, even if two different cell types or tissues had been assayed for one individual. In addition, we performed a manual curation to assign each sample to one of 41 tumor types. Next to these 15,878 tumor samples, we identified an additional 3,545 breast cancer samples, 114 esophageal cancer samples, 30 pancreas cancers and 179 HNSCCs. For the breast cancer samples data on hormone receptor (ER, PR) and HER2 status was collected including the cut-off values used to define a positive or negative receptor status. We used this information to determine the receptor status of ER, PR and HER2 according to the latest guidelines as defined by the American Society of Clinical Oncology [30]. In accordance with this guideline, for ER and PR status, we used an IHC cut-off value of 1%. Samples were considered to be HER2-positive when they reached an IHC score of 3+. In addition, samples with a HER2 IHC score of 2+ with a HER2/CEP17 ratio ≥ 2.0 were also considered positive. A HER2 IHC score of +1 or 0 was considered negative. If we could not redefine the receptor status according to the ASCO guidelines, we explored the empirical expression distributions (regular mRNA expression and FGmRNA expression) for ER, PR and HER2 receptor status of negative and positive breast cancer samples (receptor status defined according to guidelines). Both ER and PR positive and negative status was best discriminated by the regular mRNA expression levels of the Affymetrix probe 205255_at. HER2 positive and negative receptor status was best discriminated by the FGmRNA expression level of Affymetrix probe 216836_s_at. We used these probes to infer receptor status of samples that were missing ER, PR or HER2 status according to guidelines. Thresholds were defined by selecting the (FG)mRNA expression value that resulted in the optimal balance in sensitivity between the reported guideline negative and positive samples. In addition, we collected all available molecular sub-classifications (normal-like, basal, luminal A, luminal B and Her2) for the breast cancer samples [30–32]. For the TBNC samples we determined the sub-classification according to Lehmann *et al*. (basal-like 1, basal-like 2, mesenchymal, mesenchymal stem-like, immunomodulatory and luminal androgen receptor) [33].

Finally, we applied FGM profiling to determine the FGM-landscape in these 19,746 tumor samples.

### Predicting *MSLN* amplification rates

For *MSLN* we quantified the percentage of samples across 41 tumor types with a significantly increased FGM-signal (*i.e.* proxy for underlying gene amplification). The threshold (except for breast cancer, pancreas cancer, esophageal cancer and HNSCC) was defined in 18,713 FGM-profiles of non-cancer samples by calculating the 97.5th percentiles for the FGM signal of *MSLN*. For breast cancer, pancreas cancer, esophageal cancer and HNSCC we used tissue type matched healthy samples (172, 77, 47 and 277 samples, respectively) to determine the 97.5^th^ percentiles for the FGM signal of *MSLN*.

For each of the 19,746 tumor samples, *MSLN* was marked as significantly amplified when the FGM-signal was above the 97.5th percentile threshold as defined in the non-cancer samples.

### Literature search

To compare the data obtained with FGM profiling with IHC data in literature, PubMed was searched for articles published in English during the period 1996 until January 2015. The following search terms were used: ‘mesothelin’, ‘expression’, ‘cancer’ and ‘tumor’ in various combinations. The articles that were found were screened for presence of IHC staining's of patient derived tumor tissue. Subsequently, numbers of tumor samples assessed and percentages of tumor samples that were called MSLN “positive” by IHC were recorded per tumor type per article. MSLN positivity was decided to be present when it was determined as positive in the original article.
